# Optimal flip angles for in vivo liver 3D *T*_1_ mapping and  *B*_1+_ mapping at 3T

**DOI:** 10.1002/mrm.29683

**Published:** 2023-05-01

**Authors:** Gabriela Belsley, Damian J. Tyler, Matthew D. Robson, Elizabeth M. Tunnicliffe

**Affiliations:** ^1^ Oxford Centre for Clinical Magnetic Resonance Research, Division of Cardiovascular Medicine, Radcliffe Department of Medicine University of Oxford Oxford UK; ^2^ Perspectum Oxford UK

**Keywords:** $B_{1}^{+}$ mapping, liver, optimal flip angles, $T_{1}$ mapping, $T_{1}$ precision

## Abstract

**Purpose:**

The spoiled gradient recalled echo (SPGR) sequence with variable flip angles (FAs) enables whole liver $T_{1}$ mapping at high spatial resolutions but is strongly affected by $B_{1}^{+}$ inhomogeneities. The aim of this work was to study how the precision of acquired $T_{1}$ maps is affected by the $T_{1}$ and $B_{1}^{+}$ ranges observed in the liver at 3T, as well as how noise propagates from the acquired signals into the resulting $T_{1}$ map.

**Theory:**

The $T_{1}$ variance was estimated through the Fisher information matrix with a total noise variance including, for the first time, the $B_{1}^{+}$ map noise as well as contributions from the SPGR noise.

**Methods:**

Simulations were used to find the optimal FAs for both the $B_{1}^{+}$ mapping and $T_{1}$ mapping. The simulations results were validated in 10 volunteers.

**Results:**

Four optimized SPGR FAs of 2°, 2°, 15°, and 15° (TR = 4.1 ms) and $B_{1}^{+}$ map FAs of 65° and 130° achieved a $T_{1}$ coefficient of variation of 6.2 ± 1.7% across 10 volunteers and validated our theoretical model. Four optimal FAs outperformed five uniformly spaced FAs, saving the patient one breath‐hold. For the liver $B_{1}^{+}$ and $T_{1}$ parameter space at 3T, a higher return in $T_{1}$ precision was obtained by investing FAs in the SPGR acquisition rather than in the $B_{1}^{+}$ map.

**Conclusion:**

A novel framework was developed and validated to calculate the SPGR $T_{1}$ variance. This framework efficiently identifies optimal FA values and determines the total number of SPGR and $B_{1}^{+}$ measurements needed to achieve a desired $T_{1}$ precision.

## 1 INTRODUCTION

$T_{1}$ mapping is a promising non‐invasive biomarker for the diagnosis and stratification of liver diseases.^1^, ^2^, ^3^, ^4^ MOLLI $T_{1}$ values corrected for the presence of iron in the liver^5^ correlate with liver fibrosis.^1^ MOLLI is a repeatable and precise $T_{1}$ mapping method,^6^, ^7^ but has drawbacks. It offers limited liver coverage as it is a single slice breath‐hold technique. Using single‐slice $T_{1}$ mapping methods, the detection of tumors or other liver diseases affecting localized areas of the liver are likely to be missed. MOLLI acquisitions are also commonly restricted to modern scanners with access to a cardiac license. Moreover, $T_{1}$ values measured with MOLLI are biased^8^ by several factors, the largest of which are magnetization transfer^9^ and T2.^8^

The variable flip angle (VFA) spoiled gradient recalled echo (SPGR) acquisition is a widely available sequence that offers the possibility of performing whole‐liver $T_{1}$ mapping. Research using the VFA SPGR sequence in the brain for $T_{1}$ mapping has shown that its $T_{1}$ accuracy is strongly dependent on corrections for $B_{1}^{+}$ inhomogeneities.^10^, ^11^, ^12^, ^13^, ^14^ Research on the liver using this sequence is primarily restricted to contrast studies using gadoxetic acid, some of which are starting to acquire a $B_{1}^{+}$ map.^15^, ^16^, ^17^

The precision of VFA SPGR 3D $T_{1}$ mapping will be largely driven by the choice of FAs and TR for the application of interest. Deoni et al.^18^ showed that the two optimal angles straddle the Ernst angle and give a signal equal to 0.71 of the Ernst angle signal. Repeating the two optimal FAs was also shown to result in better $T_{1}$ precision compared to using a range of FAs symmetrically sampled around the Ernst angle. This early work, performed at 1.5T, did not yet include $B_{1}^{+}$ mapping for FA correction.

Given the need to acquire $B_{1}^{+}$ maps, the noise propagated from the $B_{1}^{+}$ map should also be considered when using the $T_{1}$ variance to find the optimal FAs for the VFA SPGR. For two SPGR FAs, Helms et al.^19^ derived an analytical expression to calculate $T_{1}$. Lee et al.^20^ used this expression to derive the variance in $T_{1}$, including the effect of variance from the $B_{1}^{+}$ measurement through error propagation. However, this approach is limited to two SPGR FAs. The approach of Cheng et al.^11^ is valid for an arbitrary number of FAs, but uses the linear form of the SPGR steady‐state equation, which has been shown by Chang et al.^21^ to result in $T_{1}$ overestimates of 10%–20% for whole‐brain SPGR data (1.5T, TR = 8 ms, 1 mm^3^ resolution, FAs = 2°, 3°, 14°, 17°).^21^

The Fisher information matrix allows estimating the variance in $T_{1}$ for more than two FAs using the non‐linear steady‐state equation. Lewis et al.^22^ determined the optimal SPGR FAs by minimizing a cost function given by the variance in $T_{1}$ weighted by the joint probability density of $M_{0}$ and $T_{1}$. Nataraj et al.^23^ used a min‐max Cramér‐Rao bound to find optimal FAs and TRs for precise $T_{1}$ and $T_{2}$ estimation. The optimization was carried out over a range of $T_{1}s$, $T_{2}s$ and ±10% $B_{1}^{+}$ inhomogeneities. Although the $B_{1}^{+}$ factor was included in the $T_{1}$ variance calculation, the noise in the $B_{1}^{+}$ map was not considered.

The work in this paper aims to define and validate a framework to calculate the $B_{1}^{+}$ and $T_{1}$ variance. This framework was used to find the optimal FAs for $B_{1}^{+}$ mapping and the VFA SPGR acquisition that result in a precise estimate of $T_{1}$ over a wide range of clinically relevant $T_{1}s$ and $B_{1}^{+}$ inhomogeneities typically observed in the liver at 3T. A novelty in our approach for the calculation of optimal FAs is the inclusion of $B_{1}^{+}$ uncertainties. This allowed answering the question: Does one have a higher return in $T_{1}$ precision by investing extra breath‐holds in the SPGR or the $B_{1}^{+}$ map? This enabled us to explore the number of breath‐holds required for a target $T_{1}$ precision, given knowledge of the $B_{1}^{+}$ method noise and SPGR SNR. Experimental validation of the simulations was carried out in vivo, across 10 volunteers. This framework may be useful for ultra‐high fields, where $B_{1}^{+}$ inhomogeneities extend over a larger range.

## 2 THEORY

### 2.1 $T_{1}$ mapping

$T_{1}$ mapping using the SPGR sequence is based on acquiring data at different FAs to reconstruct the SPGR curve and estimate the $T_{1}$ that best fits the data points. The SPGR signal assuming steady‐state is:

(1) $$S_{i}=M_{0}\frac{1-\exp\left(\frac{-\mathrm{TR}}{T_{1}}\right)}{1-\cos\left(\alpha_{i}\right)\exp\left(\frac{-\mathrm{TR}}{T_{1}}\right)}\sin\left(\alpha_{i}\right)$$

where $S_{i}$ is the (noiseless) signal acquired using an excitation FA of $\alpha_{i}$, $M_{0}$ is a numerical constant including the proton density, signal decay due to $T_{2}^{⋆}$ relaxation, and the $B_{1}^{-}$ receive sensitivity; TR is the repetition time, and $T_{1}$ is the longitudinal relaxation time.

### 2.2 $B_{1}^{+}$ mapping

$B_{1}^{+}$ inhomogeneities are the main source of inaccuracy in determining $T_{1}$ through Equation (1).^10^, ^11^, ^12^, ^13^, ^14^ For $B_{1}^{+}$ mapping, the ratio (R) was taken between two fully relaxed signals acquired at FA kα ($S_{k\alpha }$) and FA α ($S_{\alpha }$).^24^ The true FA exciting the spins is estimated through Equation (2) for k=2:

(2) $$\alpha \left(r\right)=\arccos\left(\left|\frac{S_{2\alpha }\left(r\right)}{2S_{\alpha }\left(r\right)}\right|\right)$$

where **r** = (*x*, *y*, *z*). The $B_{1}^{+}$ correction factor is the ratio between the true FA and the nominal FA prescribed at the scanner.

However, a non‐uniform slice profile invalidates Equation (2). To correct for slice profile effects, the complex transverse signal needs to be simulated and integrated across the slice before taking the ratio of the absolute values of the signals. A look‐up‐table based on this procedure can be used to interpolate the acquired signal ratio and find the true FA exciting the spins.^25^

### 2.3 $B_{1}^{+}$ map variance

The optimal pair of FAs (α,kα) that minimize the $B_{1}^{+}$ factor variance is found through error propagation of the noise from FAs α and kα according to Equations (3) and (4)

(3) $$\sigma_{R}=\left|R\right|*\sqrt{{\left(\frac{\sigma_{S_{k\alpha }}}{S_{k\alpha }}\right)}^{2}+{\left(\frac{\sigma_{S_{\alpha }}}{S_{\alpha }}\right)}^{2}}$$

where $\sigma_{S_{k\alpha }}$ represents the signal noise at FA kα. Once $\sigma_{R}$ is determined, Equation (4) gives an estimate of the variance associated with the $B_{1}^{+}$ map.

(4) $$\sigma_{B_{1}^{+}}^{2}={\left(\frac{\sigma_{R}}{\alpha_{\text{nominal}}{\left(\partial R/\partial \alpha \right)}_{\alpha_{\text{true}}}}\right)}^{2}$$

### 2.4 $T_{1}$ variance

The optimal SPGR FAs are selected by minimizing the variance in $T_{1}$. During the fitting of the SPGR data to Equation (1), $M_{0}$ is also an unknown parameter, making the relevant parameter vector $\theta =\left[M_{0},T_{1}\right]$. The minimum variance in $T_{1}$ is given by the Cramér‐Rao lower bound^26^ (CRLB):

(5) $$\sigma_{T_{1}}^{2}\geq {\left[{ℱ}^{-1}\left(\theta \right)\right]}_{T_{1},T_{1}}$$

where ℱ is the Fisher information matrix:

(6) $$ℱ{\left(\theta \right)}_{k,j}=-E\left[\frac{\partial^{2}\ln p\left(y,x,\theta \right)}{\partial \theta_{k}\partial \theta_{j}}\right].$$

The function p(y,x,θ) represents the likelihood function (Equation 7), which was modeled as a multivariate Gaussian with mean equal to the steady‐state signal. The steady‐state signal is a function of the independent variables of the measurement $x=\left[\mathrm{FA},\mathrm{TR},B_{1}^{+}\right]$. The vector $y=\left[y_{1},y_{2},..,y_{n}\right]$ represents the measurements acquired at each FA and fixed TR. The total number of measurements acquired is N. $\sigma_{i}$ is the total noise associated with the measurement at the *i*th FA.

(7) $$\begin{array}{cc} p\left(y,x,\theta \right) & ={\left(\frac{1}{\sqrt{2\pi }}\right)}^{N}\left(\frac{1}{\prod_{i=1}^{N}\sigma_{i}}\right)\\ & \;\exp\left[-\frac{1}{2}\sum_{i=1}^{N}\frac{1}{\sigma_{i}^{2}}{\left(y_{i}-S_{i}\left(x,\theta \right)\right)}^{2}\right]. \end{array}$$

After taking the second derivative and the expectation in Equation (6), the Fisher information matrix simplifies to:

(8) $${\left[ℱ\left(\theta \right)\right]}_{k,j}=\sum_{i=1}^{N}\;\frac{1}{\sigma_{i}^{2}}\left(\frac{\partial S_{i}}{\partial \theta_{k}}\frac{\partial S_{i}}{\partial \theta_{j}}\right).$$

To find the unknown parameters $M_{0}$ and $T_{1}$, a non‐linear least squares approach was applied. The residuals of the cost function were weighted by the inverse of the total noise variance in the measurement ($\sigma_{i}^{2}$). The total noise variance in Equation (9) has two contributions: the noise in the SPGR measurement ($\sigma^{\text{SPGR}}$) as well as the uncertainty propagated from the $B_{1}^{+}$ map measurement ($\sigma_{B_{1}^{+}}$). This latter uncertainty has been ignored in previous works.^11^, ^22^, ^23^, ^27^, ^28^

(9) $$\sigma_{i}^{2}={\left(\sigma^{\text{SPGR}}\right)}^{2}+{\left(\frac{\partial S_{i}}{\partial \alpha_{i}^{\text{true}}}\alpha_{i}^{\text{nominal}}\sigma_{B_{1}^{+}}\right)}^{2}$$

where $\alpha^{\text{true}}=B_{1}^{+}\alpha^{\text{nominal}}$. Calculating the inverse of the Fisher information matrix using Equations (8) and (9) gives a lower bound on the $T_{1}$ variance.

### 2.5 Finding the optimal FAS through a min‐max approach

Min‐max is commonly used in optimization problems to find a robust solution that is optimal for the worst‐case over a given parameter range.^29^ It finds the FA combination that results in the minimum coefficient of variation (COV) in $B_{1}^{+}$ or $T_{1}$ for the worst‐case in the parameter range. Equation (10) illustrates the min‐max approach when optimizing $T_{1}$ measurements.

(10) $$\alpha_{\text{Optimal}}=\arg\min_{\left\{\alpha \right\}}\max_{\left\{B_{1}^{+},T_{1}\right\}}{\mathrm{CoV}}_{T_{1}}\left(\alpha ,B_{1}^{+},T_{1},\mathrm{TR},\sigma_{B_{1}^{+}},\sigma_{\text{SPGR}}\right).$$

## 3 METHODS

### 3.1 Simulations: Optimal FAs for $T_{1}$ mapping

Equation (10) was implemented in a custom‐built script in MATLAB to find the optimal FAs to run in the VFA SPGR. The five main inputs to estimate the variance in $T_{1}$ are the $T_{1}$ range, TR, $B_{1}^{+}$ factor range, the associated noise in the $B_{1}^{+}$ map, the noise in the VFA SPGR and the (arbitrary) value of $M_{0}$. The optimal FAs were found by following the min‐max approach applied to the $T_{1}$ COV across the whole $T_{1}$ and $B_{1}^{+}$ parameter space. We included sets of FAs with repeated measurements at the same FA, for example $2^{\circ }$, $3^{\circ }$, $15^{\circ },$ $15^{\circ }$ is a valid four‐FA set. The maximum FA available at the scanner console was $15^{\circ }$ to achieve whole liver coverage within a 15 s breath‐hold. The code is available here: https://github.com/gabrielaBelsley/OptimalFAs_3DT1Maps.

A population at risk for non‐alcoholic fatty liver disease had MOLLI derived iron corrected values varying between 573 and 990 ms.^30^ MOLLI is known to underestimate $T_{1}$ ^8^; thus, a conversion factor given by the ratio of 812 to 666 ms was applied resulting in a $T_{1}$ range between 700 to 1200 ms at 3T.^30^, ^31^

Measurements indicated typical SPGR SNRs of 45 at $2^{\circ }$ in a healthy volunteer with low body mass index (BMI), and of 25 in a higher BMI patient with liver disease. Simulations were carried out at noise levels derived from SNRs of 12.5, 25, and 50.

The $B_{1}^{+}$ factor range was limited between 0.59 and 1.14,^32^ with 0.05 increments. An array of four different uncertainties in $B_{1}^{+}$ factor ranging from 4.6% to 2.3%, decreasing in increments of $1/\sqrt{N}$ with N = [2–4], were studied to explore the propagation of noise from the $B_{1}^{+}$ maps into the $T_{1}$ maps. The highest $B_{1}^{+}$ factor uncertainty of 4.6% was the worst‐case across 10 volunteers, corresponding to an SPGR SNR of 19 (nominal $\mathrm{FA}=2^{\circ }$, TR = 4.1 ms). Given we adopted a min‐max approach, a non‐homogenous distribution of the $B_{1}^{+}$ uncertainty was not modeled. The $B_{1}^{+}$ uncertainty represents the noise of the $B_{1}^{+}$ factor that results in the worst‐case COV $T_{1}$ across the $B_{1}^{+}$ and $T_{1}$ parameter space.

### 3.2 Simulations: Optimal FAs for the $B_{1}^{+}$ mapping

The optimal $B_{1}^{+}$ FAs are the ones that minimize the uncertainty in $B_{1}^{+}$. The uncertainty was calculated using an error propagation approach and validated through Monte Carlo (MC) simulations. The SNR used for both simulations was 12 at a true FA of $65^{\circ }$. This corresponded to the 25th quantile of the in vivo SNRs measured in the liver, across 10 healthy volunteers. For each nominal FA pair (α,kα), the $B_{1}^{+}$ factor standard deviation (SD) was calculated over a range of $B_{1}^{+}$ inhomogeneities (0.59–1.15, steps of 0.05)^32^ and k factors (1.25–4, steps of 0.25). The signals were Bloch simulated^33^ for a range of FAs ($1^{\circ }-360^{\circ }$, steps of $1^{\circ }$) taking into account slice profile effects of the RF pulse. Using a min‐max approach, the optimal FA pair was the one that achieved the minimum $B_{1}^{+}$ factor uncertainty for the true $B_{1}^{+}$ factor that yielded the highest $B_{1}^{+}$ factor SD.

In the MC simulations, for each nominal FA pair and $B_{1}^{+}$ factor, 10 000 iterations were used to calculate the ratio and estimate the $B_{1}^{+}$ factor SD. The complex Bloch simulated signals were corrupted with zero mean additive complex Gaussian noise, corresponding to an SNR of 12 at a true FA of $65^{\circ }$. The estimated FA was found by matching the ratio of the noisy signals with an interpolated noise‐free off‐line ratio, calculated over the k range and true FAs up to the ambiguity angle ($95^{\circ }$ for our RF pulse). Beyond the ambiguity angle, the function corresponding to the ratio of the signals at kα and α is no longer injective (Supporting Information Figure S1). The $B_{1}^{+}$ factor deviation is the difference between the estimated $B_{1}^{+}$ factor and the true $B_{1}^{+}$ factor.

### 3.3 Image acquisition and processing

To validate the simulations, imaging data were acquired from 10 healthy volunteers, 5 male and 5 female, on a 3T Siemens Prisma (Siemens Healthineers, Erlangen, Germany) scanner. Volunteers were scanned according to our institution's ethical practices.

A 2D multi‐slice gradient recalled echo (GRE) EPI sequence was used for the $B_{1}^{+}$ mapping with fat saturation and nominal FAs of $65^{\circ }$ and $130^{\circ }$. Acquisition parameters were: $\mathrm{FOV}=450\times 366\;mm^{2}$, matrix=64×52, slice thickness of 8 mm, gap of 2 mm, 15 slices, TE/TR = 11/10 000 ms without acceleration. The bandwidth (BW) was 3906 Hz/pixel to achieve a minimum echo spacing of 0.3 ms and the slices were acquired in an interleaved scheme. Each FA was acquired during a 10 s breath‐hold.

A 2D multi‐slice double echo spoiled GRE acquisition with magnitude and phase reconstructed data was acquired to compute a $B_{0}$ map. The $B_{0}$ map was used for distortion correction of the GRE‐EPI images through *fsl fugue* ^34^, ^35^ and modeling of $B_{0}$‐variations through slice in the $B_{1}^{+}$ map calculation. The TEs were 4.78 and 7.17 ms, TR = 20 ms, $\mathrm{FA}=15^{\circ }$, $\mathrm{FOV}=450\times 380\;mm^{2}$, matrix=64×54, slice thickness of 8 mm, gap of 2 mm, 15 slices, monopolar readout gradients, BW = 630 Hz/pixel, GRAPPA^36^ with two times acceleration in the phase encoding direction. Data were acquired during a single breath‐hold of 8.6 s.

The $T_{1}$ contrast of the liver tissue was obtained through a 3D VFA SPGR with DIXON^37^ fat/water separation. Acquisition parameters were: $\mathrm{FOV}=450\times 366\times 144\;mm^{3}$, matrix=320×260×48, TR/TEs = 4.1/[1.23, 2.46] ms, BW = 1040 Hertz/pixel. For an SNR of 12.5, the four optimal FAs that minimized the worst‐case $T_{1}$ COV were $2^{\circ }$, $3^{\circ }$, $15^{\circ },$ $15^{\circ }$. However, to calculate the SPGR SNR in vivo, a repetition at the lowest FA (where there is no contrast from vessels) was needed. Hence, it was decided to use FAs of $2^{\circ }$, $2^{\circ }$, $15^{\circ }$, $15^{\circ }$ in vivo, hereafter referred to as the standard four FAs. The standard two FAs set was $2^{\circ }$, $15^{\circ }$; the three FAs set was $2^{\circ }$, $2^{\circ }$, $15^{\circ }$ and the five FAs set was $2^{\circ }$, $2^{\circ },2^{\circ }$, $15^{\circ }$, $15^{\circ }$. Uniformly spaced FAs of $3^{\circ }$, $6^{\circ }$, $9^{\circ }$, $12^{\circ }$, $15^{\circ }$ (starting from the maximum FA of $15^{\circ }$ achieved at our scanner and decreasing in increments of $3^{\circ }$) were also acquired. Caipirinha^38^ was used with an acceleration factor of three along the slice direction with 24 separate GRE reference lines. Spatial saturation was turned off as it perturbed the theoretical steady‐state signal. Each FA is acquired independently in a breath‐hold of 15 s.

The $B_{1}^{+}$ map was calculated with a correction for slice profile effects and off‐resonance variations through slice. A non‐linear least squares fit was used to find the $B_{1}^{+}$ correction factor corresponding to the simulated ratio that best matched the ratio between the distortion corrected GRE‐EPI images acquired at nominal FAs of $130^{\circ }$ and $65^{\circ }$. The simulated ratio was computed as follows: the transverse signals at each FA were Bloch simulated^33^ across the slice direction, including the off‐resonance at each slice position extrapolated from the $B_{0}$ map.^39^ The complex transverse signal immediately after the RF pulse was propagated until time TE including free precession at the corresponding off‐resonance. The complex signals at time TE were integrated across the slice dimension.

The $B_{1}^{+}$ map was linearly interpolated to the SPGR spatial resolution. The interpolated $B_{1}^{+}$ factor was multiplied by the nominal FAs to obtain the true FAs. A correction for incomplete spoiling was applied to the SPGR signal. The correction used extended phase graph simulations^40^ to re‐scale the signal to the theoretical steady‐state value. The signal was then fit to the steady‐state SPGR function through a non‐linear least squares regression using MATLAB's^41^ function *lsqnonlin*. Repeated FAs were not averaged prior to fitting to avoid propagating any respiratory misalignments to the average image.

### 3.4 Experimental in vivo precision of the $T_{1}$ maps and $B_{1}^{+}$ maps

$T_{1}$ maps were analyzed by placing three circular regions of interest (ROIs) per slice, each with a radius of four pixels. The locations of these ROIs were chosen in the FA $15^{\circ }$ SPGR image in vessel and bile free areas, avoiding the edges of the liver.

A weighted mean $T_{1}$ and SD were calculated for each subject from the $T_{1}s$ extracted from each ROI selected in each slice. The weights for each ROI were given by the inverse of the standard error in the mean squared.

The acquisition of two $B_{1}^{+}$ maps, for each subject, enabled the calculation of the $B_{1}^{+}$ map precision. The three ROIs selected in each slice of the SPGR acquisition were applied to the two $B_{1}^{+}$ maps interpolated to the SPGR resolution. The mean $B_{1}^{+}$ factor was calculated for each ROI. A histogram of the liver‐only portion of the $B_{1}^{+}$ maps was constructed with $B_{1}^{+}$ bins of width equal to 0.1. Using the whole liver provides enough pixels to adequately calculate the uncertainty compared to just using the pixels in each ROI. Moreover, bins were used as the $B_{1}^{+}$ factor uncertainty decreases with the $B_{1}^{+}$ factor value (Supporting Information Figure S2). The $B_{1}^{+}$ factor uncertainty was calculated by taking the SD of pixel‐wise differences between the two binned $B_{1}^{+}$ maps and dividing by the square root of two. The $B_{1}^{+}$ factor uncertainty for each ROI was given by the $B_{1}^{+}$ factor SD of the bin containing the mean $B_{1}^{+}$ factor of the ROI.

### 3.5 Prediction of in vivo $T_{1}$ precision using the CRLB

After acquiring the data, the simulations were run again to compare the CRLB derived $T_{1}$ COV against the experimental weighted $T_{1}$ COV for each subject. An estimate for the $T_{1}$ variance was calculated for each ROI. The $M_{0}$ and $T_{1}$ were set to the experimental mean value within each ROI. The mean $B_{1}^{+}$ factor and the associated uncertainty for each ROI were calculated as described in Section 3.4. The noise in the SPGR was calculated for each ROI from the difference between two in vivo images obtained under identical conditions at a nominal FA of $2^{\circ }$ for the VFA SPGR, chosen to minimize the effect of vessels. A final $T_{1}$ variance for each subject was calculated from the weighted ROI $T_{1}$ variances (Supporting Information).

(11) $$w\sigma_{T_{1},\text{Simulations}}=\sqrt{\sum_{r=1}^{N_{\text{ROIs}}}w_{r}\left(\sigma_{r}^{2}+{\left(\mu_{r}-w\mu_{\text{Subject}}\right)}^{2}\right)}$$

## 4 RESULTS

### 4.1 Simulations: Optimal FAs for $T_{1}$ mapping

**FIGURE 1:**
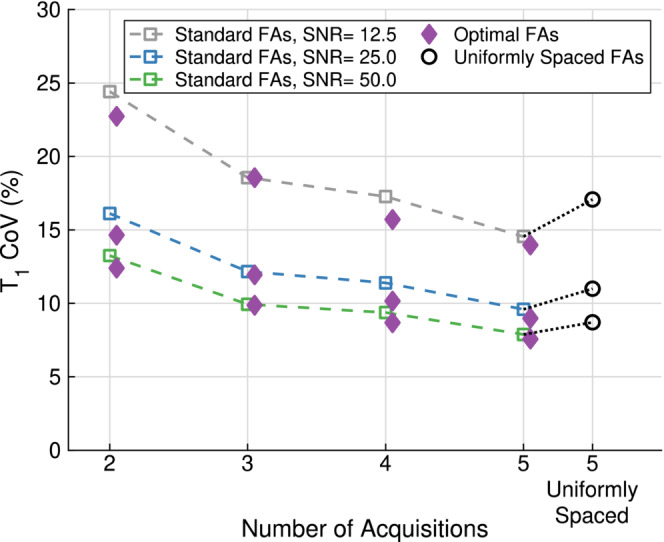
$T_{1}$ coefficient of variation (COV) as a function of the number of acquisitions (flip angles [FAs]) used to estimate $T_{1}$ for three different spoiled gradient recalled echo (SPGR) SNRs of 12.5 (gray), 25 (blue), and 50 (green), at the largest $B_{1}^{+}$ factor SD of 4.6%. Standard FAs (hollow squares) are compared against optimal FAs (purple diamonds) and five uniformly spaced FAs (black hollow circles). The standard two FAs set was $2^{\circ }$, $15^{\circ }$; the three FAs set was $2^{\circ }$, $2^{\circ }$, $15^{\circ }$; the four FAs set was $2^{\circ }$, $2^{\circ }$, $15^{\circ }$, $15^{\circ }$; and the five FAs set was $2^{\circ }$, $2^{\circ },2^{\circ }$, $15^{\circ }$, $15^{\circ }$. The optimal FAs for each SNR are found in Table 1 and the five uniformly spaced FAs were $3^{\circ }$, $6^{\circ },9^{\circ }$, $12^{\circ }$, $15^{\circ }$.

Figure 1 shows how the precision varies with the total number of acquisitions for three SNR cases. Using four optimal FAs resulted in a lower $T_{1}$ COV compared to five uniformly spaced FAs of $3^{\circ }$, $6^{\circ }$, $9^{\circ }$, $12^{\circ }$, $15^{\circ }$. The $T_{1}$ COV for the standard FAs was comparable to that of the optimal FAs.

**FIGURE 2:**
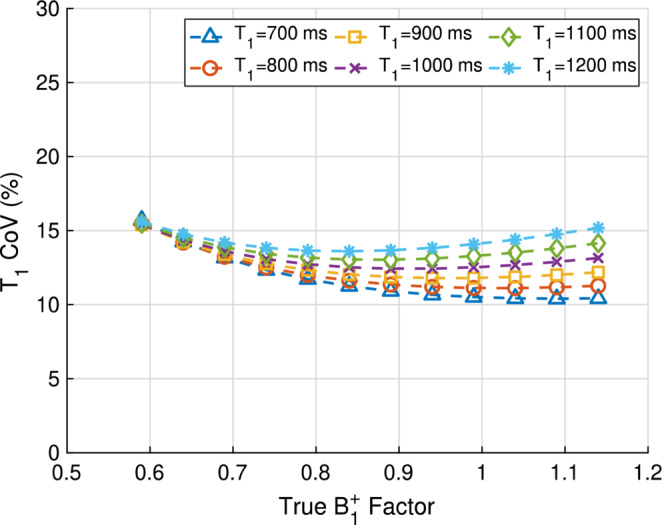
Simulated $T_{1}$ coefficient of variation (COV) as a function of $T_{1}$ and $B_{1}^{+}$ parameter space for four optimal flip angles (FAs) with a $B_{1}^{+}$ factor SD of 4.6%, and an SNR of 12.5 measured at a true FA of $2^{\circ }$, TR = 4.1 ms, $T_{1}=800\;\mathrm{ms},$ and $M_{0}=5000$. The smallest $B_{1}^{+}$ factor of 0.59 and the smallest $T_{1}$ of 700 ms were responsible for the largest $T_{1}$ COV of 15.7%. At the lowest $B_{1}^{+}$ factor, the $T_{1}$ COV is nearly independent of $T_{1}$, whilst for the largest $B_{1}^{+}$ factor the $T_{1}$ COV increases with $\;T_{1}$.

Figure 2 shows how the precision varies for the different $T_{1}s$ and $B_{1}^{+}$ factors that can be measured in vivo in the liver at 3T. The worst‐case $T_{1}$ imprecision corresponded to the lowest $B_{1}^{+}$ factor (0.59) and the lowest $T_{1}$ value (700 ms). The FA set $2^{\circ }$, $3^{\circ }$, $15^{\circ }$, $15^{\circ }$ provided the minimum $T_{1}$ COV for this worst‐case scenario. As the $T_{1}$ increases, the minimum $T_{1}$ COV is reached at $B_{1}^{+}$ factors less than 1 as the maximum sensitivity of the signal to $T_{1}$ occurs at lower FAs compared to lower $T_{1}$ values.

Table 1 shows the simulated $T_{1}$ COV obtained when using an optimal set of two, three, four, and five FAs, together with the standard FA set, using the min‐max approach across a $T_{1}$ range of 700 to 1200 ms and a $B_{1}^{+}$ factor varying between 0.59 and 1.14 with a constant $B_{1}^{+}$ factor uncertainty of 4.6%, for three different SNRs.

**TABLE 1:** $T_{1}$ coefficient of variation (COV) for the optimal and standard set of 2, 3, 4, and 5 acquisitions, for three different SNR values of 12.5, 25, and 50 (measured at a true flip angle [$\mathrm{FA}]=2^{\circ }$, TR = 4.1 ms, $T_{1}=800\;\mathrm{ms}$, and $M_{0}=5000$) and the largest $B_{1}^{+}$ factor SD of 4.6%

No. of acquisitions	SNR	Optimal FA set (°)	$T_{1}$ COV (%) Optimal FA set	$T_{1}$ COV (%) Standard FA set
2	12.5	[3 13]	22.7	24.4
	25	[3 15]	14.6	16.1
	50	[4 15]	12.4	13.3
3	12.5	[2 2 15]	18.6	18.6
	25	[3 3 13]	11.9	12.2
	50	[3 3 14]	9.9	9.9
4	12.5	[2 3 15 15]	15.7	17.3
	25	[3 4 15 15]	10.2	11.4
	50	[4 5 15 15]	8.7	9.4
5	12.5	[2 2 4 15 15]	14.0	14.5
	25	[3 3 3 15 15]	9.0	9.6
	50	[3 3 4 15 15]	7.6	7.9

*Note*: The $T_{1}$ COV for each case corresponds to the worst‐case scenario within a $T_{1}$ parameter space varying between 700 and 1200 ms and a $B_{1}^{+}$ factor varying between 0.59 and 1.14.

**FIGURE 3:**
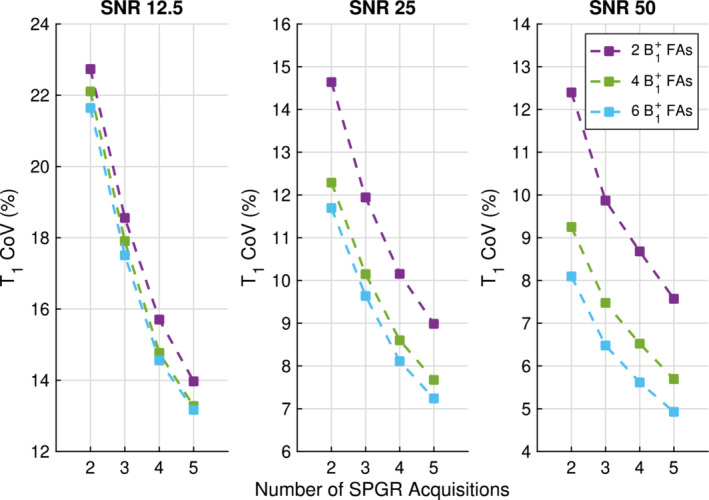
Decrease in $T_{1}$ coefficient of variation (COV) by investing acquisitions with optimal flip angles (FAs) in the spoiled gradient recalled echo (SPGR) (x axis) compared to investing two FAs (purple), four FAs (green), and six FAs (blue) in the $B_{1}^{+}$ map acquisition. The left, middle and right plots correspond to SPGR SNRs of 12.5, 25 and 50, respectively. There is almost always a higher return in $T_{1}$ COV when increasing the number of SPGR acquisitions compared to the number of $B_{1}^{+}$ map FAs. It is only best to invest in $B_{1}^{+}$ FAs for the case of three SPGR acquisitions and 2 $B_{1}^{+}$ FAs at the highest SNR. SNRs correspond to a true $\mathrm{FA}=2^{\circ }$, TR = 4.1 ms, $T_{1}=800\;\mathrm{ms}$, and $M_{0}=5000$. The $B_{1}^{+}$ factor noise for two FAs was equal to the worst‐case $B_{1}^{+}$ factor noise of 4.6%, for four FAs was 3.3% and for 6 FAs was 2.7%.

Figure 3 shows that a larger decrease in COV $T_{1}$ is achieved by investing acquisitions in SPGR compared to investing acquisitions in the $B_{1}^{+}$ map, for the 3T liver parameter space explored. For example, at an SNR of 25 starting with 2 SPGR and 2 $B_{1}^{+}$ map FAs (14.6%), adding one more SPGR acquisition leads to a better precision (11.9%) than investing two acquisitions in the $B_{1}^{+}$ map (12.3%).

### 4.2 Simulations: Optimal FAs for the $B_{1}^{+}$ mapping

An FA pair of $65^{\circ }/130^{\circ }$ minimized the uncertainty in the $B_{1}^{+}$ factor for the worst‐case $B_{1}^{+}$ inhomogeneity of 0.59, confirming the optimal k factor equals 2. The uncertainty in $B_{1}^{+}$ factor decreased as the $B_{1}^{+}$ factor increased (Figure S2). $130^{\circ }$ is the maximum FA possible at our scanner.

MC simulations confirmed the theoretical $B_{1}^{+}$ factor uncertainty from error propagation (Equation 4). For an in vivo SNR of 12 (nominal FA of $65^{\circ }$), the $B_{1}^{+}$ factor SD was 0.158 from the MC simulations and 0.159 from the error propagation, for the worst‐case $B_{1}^{+}$ factor of 0.59.

**FIGURE 4:**
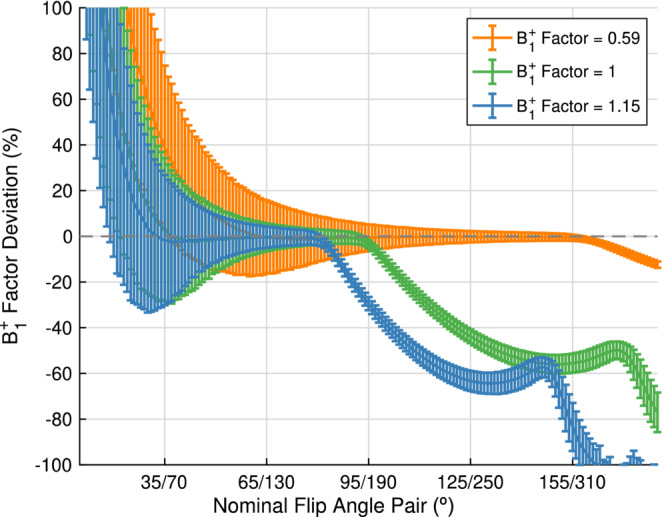
Percentage deviation in the $B_{1}^{+}$ factor estimate as a function of nominal flip angles (FA) pair for a $B_{1}^{+}$ factor of 0.59 (orange), a $B_{1}^{+}$ factor of 1 (green), and a $B_{1}^{+}$ factor of 1.15 (blue). Data from Monte Carlo simulations with 10 000 iterations. The $B_{1}^{+}$ factor error is above 2% for nominal FAs of $58^{\circ }/116^{\circ }$ or lower, at a $B_{1}^{+}$ factor of 0.59 due to low SNR. The $B_{1}^{+}$ factor error also deviates from 0% once the nominal FA pairs reach the ambiguity angle (function is no longer injective), which is reached first for a $B_{1}^{+}$ factor of 1.15 at a nominal FA pair of $83^{\circ }/166^{\circ }$. The maximum $B_{1}^{+}$ factor SD one can measure is 1.46 (95°/65°).

Figure 4 shows the deviation of the estimated $B_{1}^{+}$ correction from the true $B_{1}^{+}$ factor for each pair of FAs, for the $B_{1}^{+}$ factors of 0.59, 1.0 and 1.15. The FA lower bound is influenced by the SNR, while the upper boundary is determined by the lack of phase data from this product sequence and the magnitude of the $B_{1}^{+}$ factor inhomogeneity.

### 4.3 Experimental in vivo precision of the $T_{1}$ maps

**FIGURE 5:**
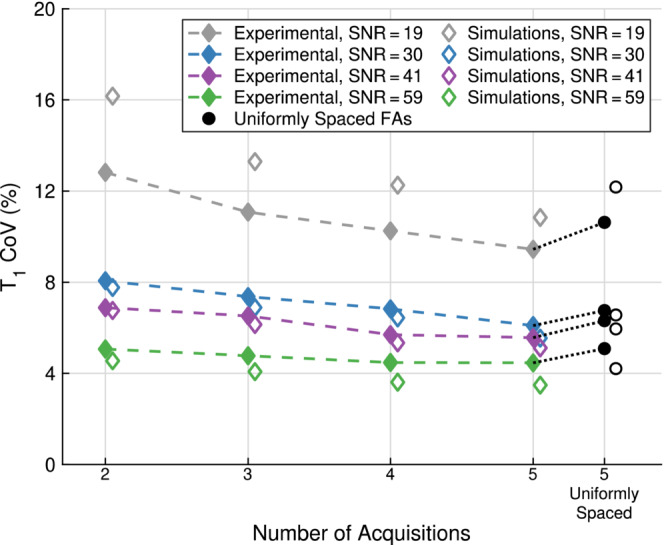
Experimental $T_{1}$ coefficient of variation (COV) as a function of the total number of acquisitions (standard flip angles [FAs]) used to estimate $T_{1}$ for four volunteers with different SPGR SNRs of 19 (gray), 30 (blue), 41 (purple) and 59 (green). The standard FAs in sets of 2, 3, 4, and 5 total FAs were ${\left[2,15\right]}^{\circ }$, ${\left[2,2,15\right]}^{\circ }$, ${\left[2,2,15,15\right]}^{\circ }$, and ${\left[2,2,3,15,15\right]}^{\circ }$, respectively. The results using five uniform FAs of ${\left[3,6,9,12,15\right]}^{\circ }$ are depicted in black. The filled markers represent the experimental $T_{1}$ COV, while the hollow markers, adjacent to the filled markers, correspond to the simulated $T_{1}$ COV using the experimental $M_{0}$, $T_{1}$, $B_{1}^{+}$ factor, spoiled gradient recalled echo (SPGR) SNR and $B_{1}^{+}$ factor SD obtained in vivo per regions of interest (ROIs).

Figure 5 shows how the $T_{1}$ COV varied in vivo as the number of standard FAs increased from two to five. A close agreement existed between the $T_{1}$ COV obtained in vivo and the $T_{1}$ COV simulated using the proposed algorithm, except for the lowest SNR.

The average SPGR SNR, $B_{1}^{+}$ map noise, mean $T_{1}$ and mean $B_{1}^{+}$ factor are shown in Table 2 for each volunteer. A large range of SNRs was observed from 19 to 59 and the mean $B_{1}^{+}$ map noise varied between 0.8% and 4.6%. The mean $T_{1}$ and $B_{1}^{+}$ factors varied between 775 and 1050 ms and 0.62 and 0.96, respectively. All these values, except SNRs above 50, are within the parameter space range used in the simulations.

**TABLE 2:** Characterization of spoiled gradient recalled echo (SPGR) SNR, $T_{1}$, $B_{1}^{+}$ factor, and $B_{1}^{+}$ factor noise parameter space for each volunteer

Volunteer	SPGR SNR	$T_{1}$ (ms) mean [min, max]	$B_{1}^{+}$ Factor mean [min, max]	$B_{1}^{+}$ Noise mean [min, max]
1	44	809 [619, 1070]	0.914 [0.691, 1.077]	0.028 [0.009, 0.099]
2	30	822 [621, 1054]	0.713 [0.521, 0.846]	0.012 [0.002, 0.055]
3	52	1050 [743, 1319]	0.937 [0.764, 1.020]	0.030 [0.011, 0.074]
4	51	947 [795, 1177]	0.892 [0.765, 1.101]	0.016 [0.004, 0.063]
5	59	985 [853, 1230]	0.959 [0.761, 1.086]	0.012 [0.004, 0.027]
6	34	775 [594, 1007]	0.809 [0.529, 1.003]	0.022 [0.008, 0.041]
7	41	793 [603, 978]	0.922 [0.699, 1.164]	0.016 [0.007, 0.037]
8	19	926 [626, 1341]	0.621 [0.474, 0.741]	0.046 [0.023, 0.079]
9	26	798 [607, 1091]	0.836 [0.619, 0.998]	0.026 [0.012, 0.044]
10	53	970 [802, 1157]	0.927 [0.723, 1.064]	0.008 [0.002, 0.020]

*Note*: The SNR is calculated from the difference of two distinct SPGR images acquired at a nominal FA of $2^{\circ }$. The mean, minimum, and maximum are reported for the $T_{1}$, $B_{1}^{+}$ factor, and $B_{1}^{+}$ factor noise. The mean values are weighted by one over the squared standard error of the mean $T_{1}$ within each region of interest (ROI).

**FIGURE 6:**
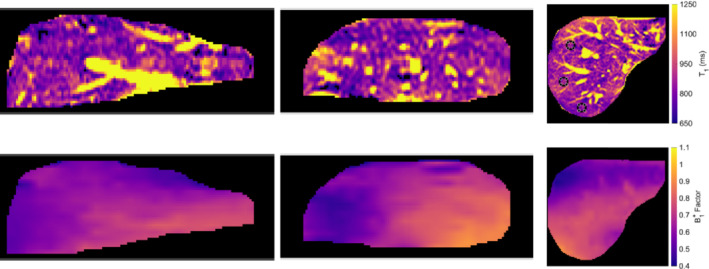
Coronal, sagittal, and axial views (with the three regions of interest [ROIs] in black) of the 3D $T_{1}$ map (first row) and $B_{1}^{+}$ factor map (second row) for one healthy volunteer. The vertical direction in the coronal and sagittal views corresponds to the number of slices. $T_{1}$ map scale for all views between 650 and 1250 ms. $B_{1}^{+}$ factor map scale for all views between 0.4 and 1.1. Supporting information shows coronal views of the 3D $T_{1}$ maps for the remaining nine volunteers.

An example of the 3D $T_{1}$ and $B_{1}^{+}$ maps in vivo is shown in Figure 6.

**FIGURE 7:**
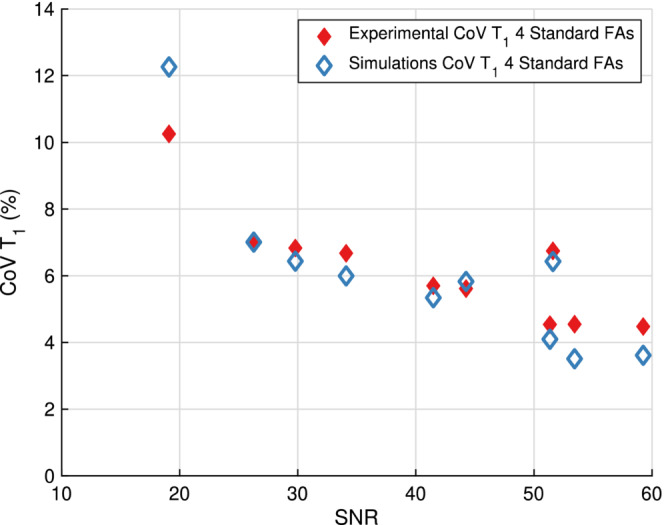
Experimental $T_{1}$ coefficient of variation (COV) as a function of SNR for the 10 volunteers. The experimental $T_{1}$ COV is shown in filled red diamonds, and the simulated $T_{1}$ COV in hollow blue diamonds for the four standard flip angles (FAs) of [2, 2, 15, 15] degrees. As the SNR increases, the $T_{1}$ COV generally decreases both experimentally and using the developed simulations algorithm. The simulated $T_{1}$ COV is in close agreement with the experimental $T_{1}$ COV.

Figure 7 shows the $T_{1}$ COV experimentally decreased as the SNR increased, illustrating the large range of SNRs in the cohort of imaged volunteers. The COV in $T_{1}$, averaged across the 10 volunteers, was 6.2±1.7%.

**FIGURE 8:**
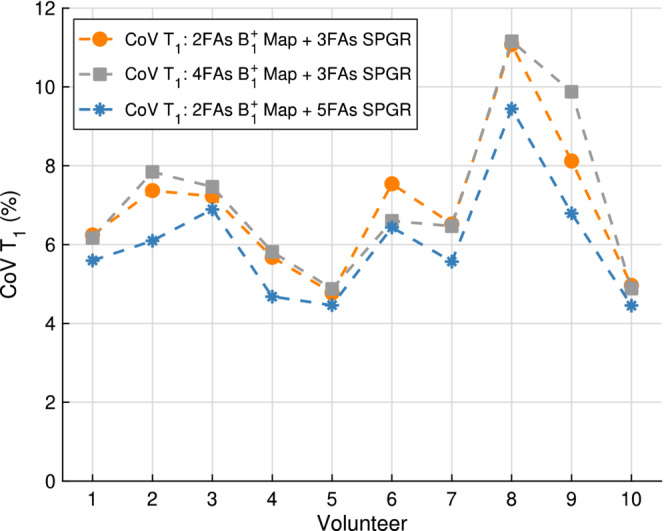
Experimental $T_{1}$ coefficient of variation (COV) using as a reference case two flip angles (FAs) in the $B_{1}^{+}$ map and three standard FAs in the spoiled gradient recalled echo (SPGR) acquisition (orange). Investing two more FAs in the $B_{1}^{+}$ map results in the gray curve, while investing two more FAs in the SPGR acquisition gives the blue curve. The in vivo data confirm the conclusions drawn from the simulation results (Figure 3) that it is best to invest FAs in the SPGR acquisition rather than the $B_{1}^{+}$ map. This occurs within the parameter space explored for all 10 volunteers.

The open research question of whether a larger decrease in $T_{1}$ COV is obtained by investing extra breath‐holds in the $B_{1}^{+}$ acquisition or the SPGR acquisition was answered in vivo by comparing the curves shown in Figure 8. It was always better to add extra breath‐holds to the SPGR acquisition for the $B_{1}^{+}/T_{1}$ parameter space observed in the liver at 3T.

## 5 DISCUSSION

While it is well known that the accuracy of the $T_{1}$ maps generated with the VFA SPGR is strongly dependent on the accuracy of the $B_{1}^{+}$ maps, what is less appreciated in the literature is how the precision of the $T_{1}$ maps are influenced by the $B_{1}^{+}$ map precision. In this work, we present a quantitative model based on the Fisher information matrix to predict the variance in $T_{1}$ considering both the noise in the SPGR acquisition and the noise in the $B_{1}^{+}$ maps. This novel model identified the optimal set of FAs for the SPGR sequence using a min‐max optimization approach on the $T_{1}$ variance across an extended range of $T_{1}s$ and $B_{1}^{+}$ factors observed in the in vivo liver at 3T. Simulations explored the robustness of the FA choice to variations in SNR and compared optimal to standard and uniformly spaced FAs. Optimizing SPGR FA choice improves $T_{1}$ map precision and can save breath‐holds. In vivo, four standard FAs resulted in better $T_{1}$ precision than five uniformly spaced FAs of ${\left[3,6,9,12,15\right]}^{\circ }$. The simulation results were validated in 10 volunteers. The close agreement between the experimental data and the simulations, within 0.5% on average, is a strong indicator that the proposed model can be used to characterize the $T_{1}$ variance and estimate optimal FAs.

The close agreement between the simulated $T_{1}$ COV, using the proposed Fisher information matrix algorithm, and the experimental data (Figure 5) for four volunteers with SNRs differing by as much as a factor of three supports the validity of our theoretical model. The largest disagreement between the experimental and simulated $T_{1}$ COV was observed for the volunteer with the lowest SNR. This volunteer, who reported falling asleep during the acquisition, had the largest $B_{1}^{+}$ map noise with a mean of 4.6%, almost 2.5 times higher than the average $B_{1}^{+}$ map noise of the other volunteers. Due to large liver misalignments (more than 1 cm between FAs was observed), the method of the differences between the two $B_{1}^{+}$ maps might have overestimated the $B_{1}^{+}$ map noise.

A rough knowledge of the in vivo SNR is helpful, but not a determining factor in finding the optimal FAs using our approach. We have shown that using the same standard FAs for all SNRs resulted in minimal increases in $T_{1}$ COV compared to the optimal FAs found for each SNR. For the liver parameter space at 3T, repeating two FAs to create a standard FA set is a robust and nearly optimal strategy. This extends Deoni's et al.^18^ conclusions for situations when $B_{1}^{+}$ inhomogeneities and uncertainties are modeled.

The strong co‐variance between $T_{1}$ and $B_{1}^{+}$ factor might motivate one to invest breath‐holds in repeating the $B_{1}^{+}$ acquisition to improve $T_{1}$ precision. However, the effect of the $B_{1}^{+}$ map noise on the $T_{1}$ map precision depends on the $B_{1}^{+}$ factor noise, the SPGR noise, $B_{1}^{+}$ factor and $T_{1}$ range. Our simulations and experimental data showed that acquiring two extra FAs in the SPGR provided a larger reduction in $T_{1}$ COV than repeating the $B_{1}^{+}$ acquisition. Our comprehensive model led to the insight that acquiring extra SPGR FAs has a double effect of reducing the uncertainties in the SPGR signal and the true FAs, while repeating the $B_{1}^{+}$ map acquisition will only reduce the noise in the $B_{1}^{+}$ map. The largest in vivo $B_{1}^{+}$ imprecision of 4.6% was used to maximize the effect of $B_{1}^{+}$ noise when evaluating where to invest the breath‐holds. Even with this conservative imprecision value, we found it's best to invest breath‐holds in the SPGR, except possibly at very high SNRs which are unlikely in liver patients.

The main motivation for carrying out this work was to provide a framework to calculate the $T_{1}$ variance including the effects of noise that propagate from the $B_{1}^{+}$ maps. This allowed us to find the minimum number of acquisitions and the FA values to guarantee a target $T_{1}$ precision, even in the worst‐case, across a wide range of $T_{1}s$ and $B_{1}^{+}s$ factors, given knowledge of the $B_{1}^{+}$ method noise and SPGR SNR. The standard set of 4 SPGR FAs ($2^{\circ }$, $2^{\circ }$, $15^{\circ }$, $15^{\circ }$) and 2 $B_{1}^{+}$ map FAs ($65^{\circ }$, $130^{\circ }$) offered a good compromise between patient comfort and the necessary $T_{1}$ precision for liver applications. Future studies will use this FA set for repeatability and clinical studies at 3T. The framework is not specific to liver applications and can be used to find the optimal compromise between the number of acquisitions and the desired precision.

### 5.1 Comparison to the literature

Both Wang et al.^42^ and Deoni et al.^18^ offered analytical expressions for the $T_{1}$ variance and emphasized the benefits of optimizing the SPGR FAs. However, their expressions are only valid for two FAs, only consider a single $T_{1}$, omit $B_{1}^{+}$ inhomogeneities and rely on the linear form of the SPGR signal. Our algorithm reproduces Deoni et al.'s optimal FAs when setting $B_{1}^{+}$ to 1, $B_{1}^{+}$ noise to 0%, using a single $T_{1}$ and the maximum FA to 20°.

Cheng et al.^11^ proposed the adoption of three angles for the SPGR extracted from two sets of optimal FA pairs using Deoni's approach, one optimal for the minimum $T_{1}$ and the other for the maximum $T_{1}$. This approach agrees with our findings that the largest $T_{1}$ COV is obtained for the extreme $T_{1}s$ (Figure 2). However, their method to calculate the $T_{1}$ variance did not consider $B_{1}^{+}$ noise in the $B_{1}^{+}$ factor measurement. Out of the four optimal FAs, the authors advise dropping one of the lowest FAs. Our results suggest that, for the narrower range of $T_{1}s$ and $B_{1}^{+}s$ found in the liver at 3T, the optimal set of three FAs uses two low FAs, which also agrees with the results of Schabel et al.^27^

The approach by Lee et al.^20^ is limited to two FAs. The authors comment in their discussion that according to their expression for $T_{1}$ variance using more than two different FAs will increase the $T_{1}$ variance. Contrary to Lee et al.^20^ our results indicate, as expected, that increasing the number of distinct FAs results in a decreased $T_{1}$ COV. In the Lee et al.^20^ formulation, the contribution due to the noise in the $B_{1}^{+}$ map is independent of the SPGR FA values. Their expression reduces to a variance in $T_{1}$ from the $B_{1}^{+}$ map noise given by $\sigma_{B_{1}^{+}}^{2}\cdot {\left(-2\cdot \mathrm{TR}\cdot \frac{T_{1}}{B_{1}^{+}}\right)}^{2}$. As a result, if one were to use Lee's algorithm to find the optimal SPGR FAs, an increase in $B_{1}^{+}$ map noise would not change the FA values. On the other hand, our CRLB algorithm includes in the weighting factor of the cost function a $B_{1}^{+}$ factor noise term that varies with the SPGR FA.

Lee et al.^20^ performed experimental measurements that suggest that the noise in the $B_{1}^{+}$ map and the noise from the individual SPGR signals, both propagated with similar weights into the $T_{1}$ map variance. For our liver parameter space at 3T, the relative contribution of the $B_{1}^{+}$ map noise into the $T_{1}$ variance varied significantly with the $B_{1}^{+}$ factor and with the SNR of the SPGR acquisition. For the lowest SNR, the $T_{1}$ variance was dominated by the SPGR noise. For the largest SNR, there was an inversion of roles with the $B_{1}^{+}$ factor noise dominating the $T_{1}$ variance over the SPGR noise.

Similar to those of Lewis et al.,^22^ our results also suggest using two angles on either side of the Ernst angle in the steeper portion of the SPGR curve. The low FA is sensitive to $M_{0}$ and insensitive to $T_{1}$ while the high FA is sensitive to $T_{1}$ variations. The authors reported higher rat ex‐vivo RMS error by 2%–3% compared to the results when using Deoni et al.'s^18^ FAs, for SNRs above 5. The authors suggested that $B_{1}^{+}$ inhomogeneities are the cause of Deoni's FAs outperforming their optimal FAs. The authors did not consider either $B_{1}^{+}$ inhomogeneities or noise during FA selection. In our case, the $T_{1}$ COV of two optimal FAs selected using Deoni's criteria^18^ increased the simulated $T_{1}$ COV by 4.2%, 2.9% and 1.9% for SNRs of 12.5, 25 and 50 respectively in our liver 3T parameter range (Supporting Information Table S2). Repeating Deoni's FA pair resulted in $T_{1}$ COV increases of 3.3%, 2.3% and 1.4% compared to the four optimal FAs found using our approach, for SNRs of 12.5, 25, and 50, respectively.

Our study shows that $B_{1}^{+}$ factors in the liver are skewed toward values below 1 (Table 2), that is, the true FA will generally be lower than the nominal FA prescribed at the scanner. This also agrees with $B_{1}^{+}$ factor values in the liver measured on GE scanners.^32^ Nevertheless, our algorithm takes the distribution of $B_{1}^{+}$ factors into account when optimizing the nominal SPGR FAs.

Our approach offers several advantages. An analytical expression characterizing the variance in $T_{1}$ was developed that considers a large range of $T_{1}$ and $B_{1}^{+}$ factors without any approximations, simplifications, or making use of the linear form of the SPGR signal. Importantly, the analytical expression is computationally inexpensive. It overcomes the time‐consuming burden of MC simulations, which can be prohibitive when including large ranges of $T_{1}$, $B_{1}^{+}$ factors and nominal FAs (in our case this resulted in a nine‐dimensional search space with five FAs). Our method applies to any research using the SPGR signal for $T_{1}$ mapping, regardless of body part imaged or $B_{1}^{+}$ mapping method adopted.

Note that our approach uses the CRLB, which assumes there is no bias in the measurement of $T_{1}$. Therefore, any errors in the $T_{1}$ due to uncompensated $B_{1}^{+}$ inhomogeneities, incomplete spoiling,^43^, ^44^ or slice profile in 2D^25^ acquisitions should be carefully considered and corrected.

## 6 CONCLUSIONS

We developed a novel theoretical framework to compute the $T_{1}$ variance that incorporates for the first time both the effect of noise from the VFA SPGR signal and the $B_{1}^{+}$ map as well as the range of $B_{1}^{+}$ factors and $T_{1}s$ observed in the liver at 3T. This framework efficiently identifies optimal FA values and determines the total number of SPGR measurements needed to achieve a certain $T_{1}$ COV threshold. Validation of several predictions of this robust framework was achieved in vivo by using the framework to optimize whole liver 3D $T_{1}$ mapping in vivo at 3T leading to an average $T_{1}$ COV of 6.2±1.7% across 10 volunteers using a total of seven breath‐holds, four standard SPGR FAs (15 s each), two $B_{1}^{+}$ FAs (10 s each) and one $B_{0}$ map FA (8.6 s).

## Supporting information


**FIGURE S1.** Ratio between the signals at FAs 2α and α, for FAs α varying between 1° and 100°. Noise in the signals results in a variation of the ratio (δR) which will correspond to a variation in the FA (δα). The larger the FA, the steeper the curve. Therefore, for a fixed uncertainty in the ratio, larger FAs yield smaller uncertainties in the $B_{1}^{+}$ factor estimate. At a FA of 95° the function is no longer injective; the non‐injectivity does not occur at 90° due to slice profile effects.
**Figure S2.** $B_{1}^{+}$ factor standard deviation as a function of $B_{1}^{+}$ factor values in the liver (at 3 T) for a nominal FA pair of (65°, 130°). The largest $B_{1}^{+}$ factor standard deviation was 0.158 and occurred for the lowest $B_{1}^{+}$ factor in the liver of 0.59. This curve was calculated using an SNR of 12 corresponding to the 25th quantile measured across 10 volunteers at a nominal FA of 65°.
**Table S1.** Comparison between $T_{1}$ CoV calculated using the CRLB and MC simulations for four optimal FAs at three different SNR levels: 12.5, 25 and 50. The SNR corresponds to a true $\mathrm{FA}=2^{\circ }$, TR = 4.1 ms, $T_{1}=800\;\mathrm{ms}$ and $M_{0}=5000$. For these calculations the min‐max approach was followed by adopting a $B_{1}^{+}$ factor of 0.59 and $T_{1}$ value of 700 ms. The $B_{1}^{+}$ factor standard deviation was 4.6%. 50 000 iterations were used for the MC simulations.
**Table S2.** Comparison between $T_{1}$ CoV obtained with the optimal set of FAs proposed and the FA set using Deoni's approach,^18^ for three different SNR values of 12.5, 25 and 50 (measured at a true $\mathrm{FA}=2^{\circ }$, TR = 4.1 ms, $T_{1}=800\;\mathrm{ms}$ and $M_{0}=5000$) and the largest $B_{1}^{+}$ factor standard deviation of 4.6%. The $T_{1}$ CoV for each case corresponds to the worst‐case scenario within a $T_{1}$ parameter space varying between 700 and 1200 ms and a $B_{1}^{+}$ factor varying between 0.59 and 1.14.
**Figure S3.** Coronal $T_{1}$ maps for the 10 healthy volunteers showing whole liver $T_{1}$ maps. The vertical direction corresponds to the number of slices. All maps plotted with a colormap scale varying between 500 and 1500 ms.

## Data Availability

The code for the T1 variance algorithm and used for generating the simulation results (Figures 1, 2, 3 and Table 1) is available here: https://github.com/gabrielaBelsley/OptimalFAs_3DT1Maps.
