# Supplementary material for: Optimal flip angles for in vivo liver 3D T 1 mapping and B 1+ mapping at 3T
Source: Magn Reson Med. 2023 May 1;90(3):950–62. doi: 10.1002/mrm.29683 (PMC10952198; doi:10.1002/mrm.29683)
Supplement: Supplementary file 1 — FIGURE S1. Ratio between the signals at FAs 2α and α, for FAs α varying between 1° and 100°. Noise in the signals results in a variation of the ratio (δR) which will correspond to a variation in the FA (δα). The larger the FA, the steeper the curve. Therefore, for a fixed uncertainty in the ratio, larger FAs yield smaller uncertainties in the B1+ factor estimate. At a FA of 95° the function is no longer injective; the non‐injectivity does not occur at 90° due to slice profile effects. Figure S2. B1+ factor standard deviation as a function of B1+ factor values in the liver (at 3 T) for a nominal FA pair of (65°, 130°). The largest B1+ factor standard deviation was 0.158 and occurred for the lowest B1+ factor in the liver of 0.59. This curve was calculated using an SNR of 12 corresponding to the 25th quantile measured across 10 volunteers at a nominal FA of 65°. Table S1. Comparison between T1 CoV calculated using the CRLB and MC simulations for four optimal FAs at three different SNR levels: 12.5, 25 and 50. The SNR corresponds to a true FA=2∘, TR = 4.1 ms, T1=800ms and M0=5000. For these calculations the min‐max approach was followed by adopting a B1+ factor of 0.59 and T1 value of 700 ms. The B1+ factor standard deviation was 4.6%. 50 000 iterations were used for the MC simulations. Table S2. Comparison between T1 CoV obtained with the optimal set of FAs proposed and the FA set using Deoni's approach,18 for three different SNR values of 12.5, 25 and 50 (measured at a true FA=2∘, TR = 4.1 ms, T1=800ms and M0=5000) and the largest B1+ factor standard deviation of 4.6%. The T1 CoV for each case corresponds to the worst‐case scenario within a T1 parameter space varying between 700 and 1200 ms and a B1+ factor varying between 0.59 and 1.14. Figure S3. Coronal T1 maps for the 10 healthy volunteers showing whole liver T1 maps. The vertical direction corresponds to the number of slices. All maps plotted with a colormap scale varying between 500 and 1500 ms. [file MRM-90-950-s001.docx]

**Supporting Information**

$\boldsymbol{B}_{\boldsymbol{1}}^{\boldsymbol{+}}$ **Variance**

Figure S1 illustrates how the uncertainty in the ratio ($\delta R)$ of the signals is mapped into an uncertainty in the FA ($\delta\alpha)$. The $B_{1}^{+}$ variance in Eq. 4 (in the main text) is the square of the ratio between the uncertainty in the FA and the nominal FA. The numerator is calculated using the slope from Figure S1.

Figure S1. Ratio between the signals at FAs $2\alpha$ and $\alpha$, for FAs $\alpha$ varying between 1˚ and 100˚. Noise in the signals results in a variation of the ratio ($\delta R$) which will correspond to a variation in the FA ($\delta\alpha$). The larger the FA, the steeper the curve. Therefore, for a fixed uncertainty in the ratio, larger FAs yield smaller uncertainties in the $B_{1}^{+}$ factor estimate. At a FA of 95˚ the function is no longer injective; the non-injectivity doesn’t occur at 90˚ due to slice profile effects.

Figure S2 shows that the uncertainty in the $B_{1}^{+}$ factor decreases as the $B_{1}^{+}$ factor increases.

Figure S2. $B_{1}^{+}$ factor standard deviation as a function of $B_{1}^{+}$ factor values in the liver (at 3T) for a nominal FA pair of [65˚, 130˚]. The largest $B_{1}^{+}$ factor standard deviation was 0.158 and occurred for the lowest $B_{1}^{+}$ factor in the liver of 0.59. This curve was calculated using an SNR of 12 corresponding to the 25^th^ quantile measured across 10 volunteers at a nominal FA of 65˚.

$\boldsymbol{T}_{\boldsymbol{1}}$ **Variance**

The derivation of the expected $T_{1}$ variance given by Eq. 5 begins with defining the Fisher information matrix through Eq. 6 in the main text,

$F\left(\mathbf{\theta}\right)_{k,j}=-E\left[\frac{\partial^{2}\ln p\left(\mathbf{x},\mathbf{y},\mathbf{\theta}\right)}{\partial\theta_{k}\partial\theta_{j}}\right]$ . [6]

In Eq. 6, $E$ denotes the expectation value and $p$ represents the likelihood function of obtaining a particular set of measurement values **y**, given a set of independent variables $\mathbf{x}=\left[x_{1},x_{2}\cdots x_{N}\right]$ and parameters $\mathbf{\theta}=\left[M_{0},T_{1}\right]$ . Assuming an unbiased measurement with normally distributed errors, the likelihood function is given by a product of N normalized Gaussian functions, one for each acquisition:

$p\left(\mathbf{x}\text{,}\mathbf{y}\text{,}\mathbf{\theta}\right)=\left(\frac{1}{\sqrt{2\pi}}\right)^{N}\left(\Pi_{i=1}^{N}\sigma_{i}\right)^{-1}\exp\left[\frac{-1}{2}\sum_{i=1}^{N} \frac{\left(y_{i}-S_{i}\left(\mathbf{x},\mathbf{\theta}\right)\right)^{2}}{\sigma_{i}^{2}}\right]$. [7]

In Eq. 7, $\sigma_{i}^{2}$ is the variance associated with each of the acquisitions and $S_{i}\left(\mathbf{x},\mathbf{\theta}\right)$ is the prediction of the SPGR steady state equation for a given true FA and TR, $x_{i}$, and parameter set $\mathbf{\theta}=\left[M_{0},T_{1}\right]$. Taking the natural logarithm of $p$,

,

and carrying out derivatives sequentially, one obtains:

$$\frac{\partial\ln p\left(\mathbf{x},\mathbf{y},\mathbf{\theta}\right)}{\partial\theta_{j}}=\sum_{i=1}^{N} \frac{\left[y_{i}-S_{i}\left(\mathbf{x},\mathbf{\theta}\right)\right]}{\sigma_{i}^{2}}\frac{\partial S_{i}\left(\mathbf{x},\mathbf{\theta}\right)}{\partial\theta_{j}}$$

$\frac{\partial^{2}\ln p\left(\mathbf{x},\mathbf{y},\mathbf{\theta}\right)}{\partial\theta_{k}\partial\theta_{j}}=-\sum_{i=1}^{N} \frac{1}{\sigma_{i}^{2}}\frac{\partial S_{i}\left(\mathbf{x},\mathbf{\theta}\right)}{\partial\theta_{k}}\frac{\partial S_{i}\left(\mathbf{x},\mathbf{\theta}\right)}{\partial\theta_{j}}+\sum_{i=1}^{N} \frac{\left[y_{i}-S_{i}\left(\mathbf{x},\mathbf{\theta}\right)\right]}{\sigma_{i}^{2}}\frac{\partial^{2}S_{i}\left(\mathbf{x},\mathbf{\theta}\right)}{\partial\theta_{k}\partial\theta_{j}}$.

Finally, under the assumption that **S** is an unbiased estimator of the steady-state SPGR signal, the expectation value of the last term on the right-hand side of the above equation vanishes leaving the final result which corresponds to Eq. 8 in the main text,

$\left[F\left(\mathbf{\theta}\right)\right]_{k,j}=\sum_{i=1}^{N} \frac{1}{\sigma_{i}^{2}}\frac{\partial S_{i}\left(\mathbf{x},\mathbf{\theta}\right)}{\partial\theta_{k}}\frac{\partial S_{i}\left(\mathbf{x},\mathbf{\theta}\right)}{\partial\theta_{j}}$. [8]

**Validation of $\boldsymbol{T}_{\boldsymbol{1}}$ Variance Framework**

To validate the theoretical Cramér-Rao lower bound (CRLB) based $T_{1}$ precision, a Monte Carlo (MC) simulation was performed over 50000 iterations. In each iteration, random noise was added to the $B_{1}^{+}$ factor. SPGR signals were generated using the noisy $B_{1}^{+}$ factor at each true FA. Subsequently, these signals were corrupted with Gaussian noise, using three SNRs of 12.5, 25 and 50. The nominal FAs were equal to the 4 optimal FAs output by the CRLB. The worst-case $B_{1}^{+}$ factor of 0.59, $B_{1}^{+}$ noise of 4.6% and $T_{1}$ of 700 ms were used, consistent with the min-max approach. The fitting parameters, $M_{0}$ and $T_{1}$, were determined using a non-linear least squares fit of the weighted noisy signal to the steady-state equation. The weights are given by Eq. 8. The results of the validation are shown in Table S1.

Table S1. Comparison between $T_{1}$ CoV calculated using the CRLB and MC simulations for 4 optimal FAs at three different SNR levels: 12.5, 25 and 50. The SNR corresponds to a true FA=$2^{\circ}$, TR=4.1 ms, $T_{1}$=800 ms and $M_{0}$=5000. For these calculations the min-max approach was followed by adopting a $B_{1}^{+}$ factor of 0.59 and $T_{1}$ value of 700 ms. The $B_{1}^{+}$ factor standard deviation was 4.6%. 50000 iterations were used for the MC simulations.

| SNR | ﻿Optimal FAs (◦) | ﻿CRLB $\boldsymbol{T}_{\boldsymbol{1}}$ CoV (%) | Monte Carlo Simulations $\boldsymbol{T}_{\boldsymbol{1}}$ CoV (%) |
| --- | --- | --- | --- |
| ﻿12.5 | ﻿[2 3 15 15] | 15.70 | 15.65 |
| 25 | ﻿[3 4 15 15] | ﻿10.15 | ﻿10.16 |
| 50 | ﻿[4 5 15 15] | ﻿8.68 | ﻿8.65 |

**Comparison of our optimal FAs with Deoni’s et al.**^1^ **FAs**

| Number of FAs | SNR | Optimal FA set (˚) | $\boldsymbol{T}_{\boldsymbol{1}}$ CoV (%)  Optimal FA set | Deoni’s^1^ FA set (˚) | $\boldsymbol{T}_{\boldsymbol{1}}$ CoV (%)  Deoni’s FA set |
| --- | --- | --- | --- | --- | --- |
| 2 | 12.5 | [3 13] | 22.7 | [2 13] | 26.9 |
|  | 25 | [3 15] | 14.6 | [2 13] | 17.5 |
|  | 50 | [4 15] | 12.4 | [2 13] | 14.3 |
| 4 | 12.5 | [2 3 15 15] | 15.7 | [2 2 13 13] | 19.0 |
|  | 25 | [3 4 15 15] | 10.2 | [2 2 13 13] | 12.5 |
|  | 50 | [4 5 15 15] | 8.7 | [2 2 13 13] | 10.1 |

Table S2 shows that the $T_{1}$ CoV using the optimal FA set presented in this paper is always lower than the $T_{1}$ CoV using the FA set calculated through Deoni’s^1^ approach.

Table S2. Comparison between $T_{1}$ CoV obtained with the optimal set of FAs proposed and the FA set using Deoni’s approach^1^, for three different SNR values of 12.5, 25 and 50 (measured at a true FA=$2^{\circ}$, TR=4.1 ms, $T_{1}$=800 ms and $M_{0}$=5000) and the largest $B_{1}^{+}$ factor standard deviation of 4.6%. The $T_{1}$ CoV for each case corresponds to the worst-case scenario within a $T_{1}$ parameter space varying between 700 ms and 1200 ms and a $B_{1}^{+}$ factor varying between 0.59 and 1.14.

**3D $\boldsymbol{T}_{\boldsymbol{1}}$ Maps for all volunteers**

Figure S3. Coronal $T_{1}$ maps for the 10 healthy volunteers showing whole liver $T_{1}$ maps. The vertical direction corresponds to the number of slices. All maps plotted with a colourmap scale varying between 500ms and 1500ms.

**Overall** $\boldsymbol{T}_{\boldsymbol{1}}$ **Uncertainty from Joining** $\boldsymbol{T}_{\boldsymbol{1}}$ **Distributions from Several Regions of Interest (ROIs)**

The distribution of $T_{1}$ values within each ROI is modelled as a Normal distribution, each with a mean value $\mu_{j},$ and a standard deviation $\sigma_{j}$:

$$P_{j}\left(x\right)=\frac{1}{\sigma_{j}\sqrt{2\pi}}\exp\left[\frac{-\left(x-\mu_{j}\right)^{2}}{2\sigma_{j}^{2}}\right]$$

where $j$ is the index running over the number of ROIs.

The weighted mean value of the union of N distributions is:

$$\mu=\sum_{j=1}^{N} {w_{j}\mu}_{j}$$

The weights for each distribution are proportional to one over its variance:

$$w_{j}=\frac{(1/\sigma_{j}^{2})}{\sum_{j=1}^{N} (1/\sigma_{j}^{2})}$$

The weights are normalised, so their sum is equal to unity.

The weighted variance of the union of N distributions is:

$$\sigma^{2}=\sum_{j=1}^{N} w_{j} \int_{-\infty}^{+\infty} P_{j}\left( x \right)\left( x-\mu\right)^{2}=\sum_{j=1}^{N} w_{j} \int_{-\infty}^{+\infty} dx\frac{\left( x-\mu\right)^{2}}{\sigma_{j}\sqrt{2\pi}}exp\left[ \frac{{-\left( x-\mu_{j} \right)}^{2}}{{2\sigma}_{j}^{2}} \right]$$

To carry out the integral for each of the distributions, the substitution $s=x-\mu_{j}$ was used:

$$\begin{matrix} \int_{-\infty}^{\infty} ds \frac{\left(s+\mu_{j}-\mu\right)^{2}}{\sigma_{j}\sqrt{2\pi}}\exp\left[\frac{-s^{2}}{2\sigma_{j}^{2}}\right] =\frac{1}{\sigma_{j}\sqrt{2\pi}}\left\{\int_{-\infty}^{\infty} ds s^{2}\exp\left[\frac{-s^{2}}{2\sigma_{j}^{2}}\right] +\int_{-\infty}^{\infty} ds \left(\mu_{j}-\mu\right)^{2}\exp\left[\frac{-s^{2}}{2\sigma_{j}^{2}}\right] \right\} \\ =\frac{1}{\sigma_{j}\sqrt{2\pi}}\left\{\sqrt{2\pi}\sigma_{j}^{3}+\sqrt{2\pi}\sigma_{j}\left(\mu_{j}-\mu\right)^{2}\right\} \end{matrix}$$

The resulting weighted variance for N distributions is:

$$\sigma^{2}=\sum_{j=1}^{N} w_{j} (\sigma_{j}^{2}+{(\mu_{j}-\mu)}^{2})$$

This formula was used to calculate the variance from the N $T_{1}$ distributions (Eq. 10) corresponding to the N ROIs.

**Reference**

1. Deoni SCL, Rutt BK, Peters TM. Rapid combined T1 and T2 mapping using gradient recalled acquisition in the steady state. *Magn Reson Med*. 2003;49(3):515-526. doi:10.1002/mrm.10407
